# Protocol of a prospective multicenter randomized controlled trial of robot-assisted stereotactic lesioning in the treatment of focal drug-resistant epilepsy

**DOI:** 10.1186/s13063-023-07334-9

**Published:** 2023-06-09

**Authors:** Mingkun Gong, Ke Xu, Yongzhi Shan, Yihe Wang, Chao Zhang, Xiongfei Wang, Jian Zhou, Yuguang Guan, Tianfu Li, Guoming Luan

**Affiliations:** 1 Department of Neurosurgery, Sanbo Brain Hospital, Capital Medical University, Haidian District, No. 50, Yikesong Road, Beijing, 100093 China; 2 Department of Neurosurgery, Xuanwu Hospital, Capital Medical University, No. 45, Changchun Street, Xicheng District, Beijing, 100053 China; 3 Department of Neurosurgery, Beijing Tiantan Hospital, Capital Medical University, No.119, South 4th Ring West Road, Fengtai District, Beijing, 10007 China; 4 Department of Neurology, Sanbo Brain Hospital, Capital Medical University, Haidian District, No. 50, Yikesong Road, Beijing, 100093 China

**Keywords:** Drug-resistant epilepsy, Robot-assisted stereotactic lesioning, Randomized controlled trial

## Abstract

**Background:**

This protocol describes the design of a multicenter randomized controlled trial of robot-assisted stereotactic lesioning versus epileptogenic foci resection. Typical causes of focal epilepsy include hippocampal sclerosis and focal cortical dysplasia. These patients usually present with drug resistance and require surgical treatment. Although epileptogenic foci resection is still the most commonly used treatment for such focal epilepsy, there is increasing evidence that epileptogenic focus resection may lead to neurological impairment. The treatment of epilepsy with a robot-assisted stereotactic lesioning mainly includes two new minimally invasive surgical methods: radiofrequency thermocoagulation (RF-TC) and laser interstitial thermal therapy (LITT). Seizure-free is less likely to be achieved by these two procedures, but neurologic preservation is better. In this study, we aimed to compare the safety and efficacy of RF-TC, LITT, and epileptogenic foci resection for focal drug-resistant epilepsy.

**Methods:**

This is a multicenter, three-arm, randomized controlled clinical trial. The study will include patients older than 3 years of age with epilepsy who have had medically refractory seizures for at least 2 years and are eligible for surgical treatment with an epileptogenic focus as determined by multidisciplinary evaluation prior to randomization. The primary outcome measure is seizure outcome (quantified by seizure remission rate) at 3-month, 6-month, and 1-year follow-up after treatment. Postoperative neurologic impairment, spectrum distribution change of video electroencephalogram, quality of life, and medical costs will also be assessed as secondary outcomes.

**Trial registration:**

Chinese Clinical Trials Registry ChiCTR2200060974. Registered on June 14, 2022. The status of the trial is recruiting, and the estimated study completion date is December 31, 2024.

## Background

Epilepsy is a common disease of the nervous system causedby different causes, with spontaneous, recurrent, and unpredictable seizures as the main characteristics. It also has numerous neurobiological, cognitive, and psychosocial consequences [1]. Several epidemiological studies show that the incidence of epilepsy is 5.5–7.3‰ [2–4]. Drug therapy is the preferred treatment for epilepsy at present. However, about 30% of patients are still unable to control their seizures after continuous treatment with two or more appropriate anti-seizure medications (ASMs), which are defined as drug-resistant epilepsy (DRE) [5, 6]. In cases of drug failure, optimal seizure control usually requires neurosurgical intervention targeting the epileptic foci. Epilepsy surgery can be divided into diagnostic surgery (subdural electrode implantation, stereotactic multicontact electrode implantation, etc.), resective surgery (lesion resection, lobectomy, hemispherectomy, etc.), and palliative neuromodulation surgery (vagus nerve stimulation, deep brain stimulation, etc.) [7–9]. Patients with drug-resistant focal epilepsy can benefit from the removal or disconnection of a circumscribed brain region to achieve complete seizure control or at least reduce disabling seizures. Among carefully selected patients, the proportion of individuals who are seizure-free after surgery ranges from 50 to 80% [10, 11].

In the 1960s, Talairach and Bancaud innovatively took the anterior and posterior connections of the brain as the benchmark for homogenization, which created the stereotactic surgery technique in neurosurgery [12]. In recent years, with the rapid development of robot-assisted technology, the stability, accuracy, and economy of stereotactic surgery have made great progress. With the assistance of a robot, the implantation time of multicontact electrodes has been shortened to 3–5 min per electrode, and the accuracy of entry point, angle, and other parameters has been significantly improved; thus, the safety of surgery has been improved [13, 14]. Today, stereoelectroencephalography (SEEG) provides a unique approach to exploring the pathophysiology of epilepsy and pinpointing the epileptogenic network that no other noninvasive examination can provide. At the same time, because of the advantages of nerve function protection, stereotactic lesioning accounts for an increasing proportion of surgical treatment in major epilepsy medical centers around the world.

In the field of epilepsy surgery, stereotactic lesioning mainly includes two forms, one is SEEG-guided radiofrequency thermocoagulation(RF-TC) and the other is laser interstitial thermal therapy (LITT). RF-TC has been used for a long time, and it has been applied to several nervous system diseases such as epilepsy and trigeminal neuralgia [15]. Its application in the field of epilepsy is mainly achieved under the guidance of SEEG electrode implantation. Its effectiveness has been reported in large cohorts from several centers in Marseille, Lyon, Milan, and other European centers [16, 17]. Zhao et al. demonstrated its effectiveness in the treatment of mesial temporal lobe epilepsy by SEEG-guided RF-TC [18].

LITT is a kind of thermocoagulation surgery which is guided by magnetic resonance or stereo-electroencephalography and monitors the temperature and range of heat generated by laser in real time. It has a wide range of applications and can be used to treat a variety of diseases, including intracranial tumors and epilepsy. Curry et al. first used LITT to treat children with DRE and achieved some efficacy [19]. Since then, several clinical studies with relatively small sample sizes have reported the success of this technique in the treatment of medial temporal lobe epilepsy, hypothalamic hamartoma, periventricular nodular heterotopia, and focal cortical dysplasia [20–23].

Due to some limitations of existing studies, there is not enough evidence to prove the effectiveness of stereotactic lesioning of focal drug-resistant epilepsy. In China, there is no guideline for stereotactic lesioning in the treatment of focal drug-resistant epilepsy. In this study, we aimed to evaluate the efficacy and safety of stereotactic lesioning compared with resective surgery.

## Methods/design

### General study design

A multicenter, randomized controlled, parallel-group clinical trial will be conducted at Sanbo Brain Hospital, Beijing Tiantan Hospital, and Xuanwu Hospital. This study has been registered in the Chinese Clinical Trial Registry before the enrollment of participants (http://www.chictr.org.cn, registration number: ChiCTR2200060974).

**Fig. 1:**
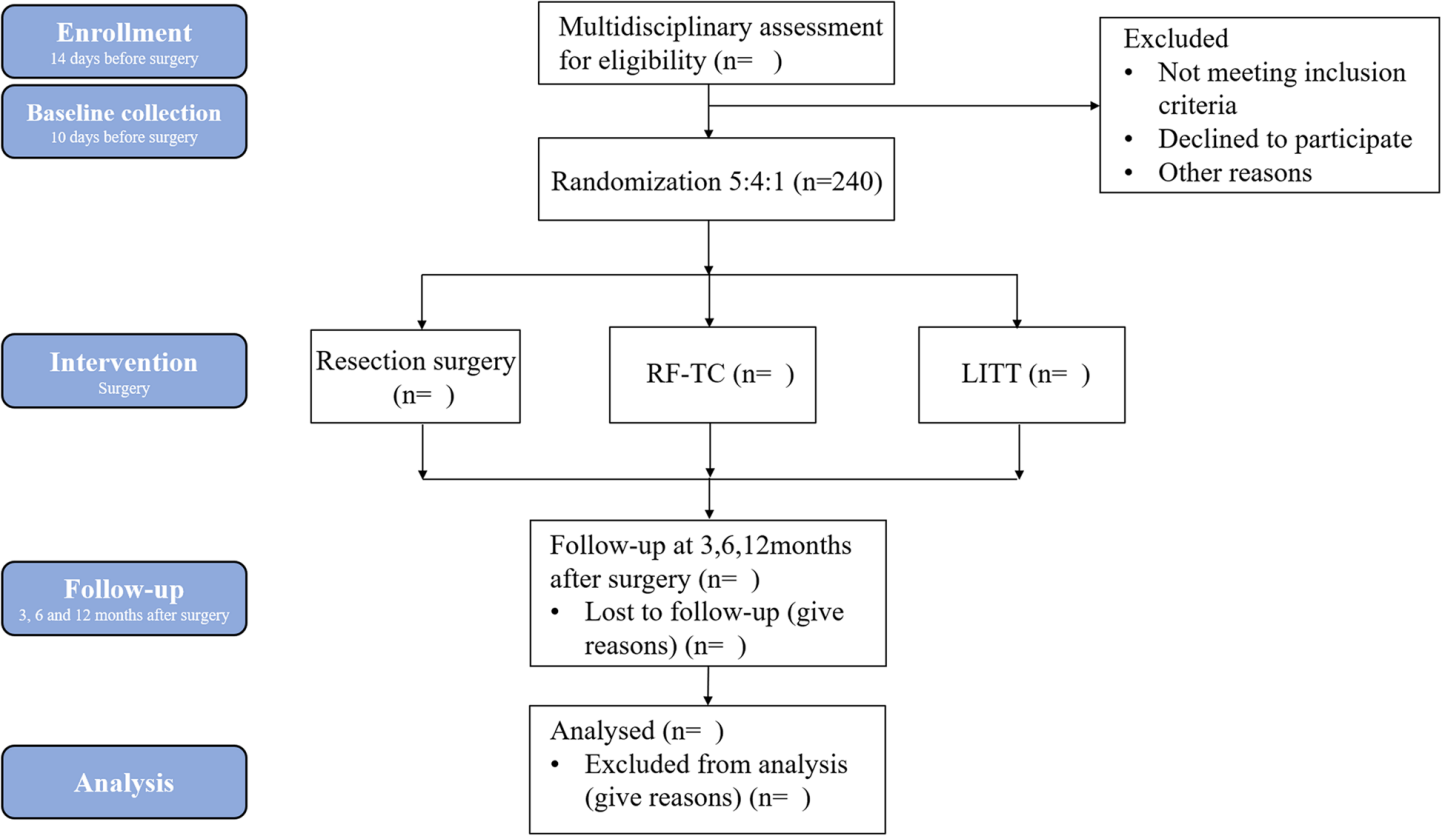
Timeline of the robot-assisted stereotactic lesioning in the treatment of focal drug-resistant epilepsy study protocol

Participant baseline information will be collected approximately 10 days before surgery, including participant demographics, seizure status, use of ASMs, neurological status, electroencephalogram (EEG), and magnetic resonance imaging (MRI) findings. The perioperative information of the participants will be recorded based on the surgical records and anesthesia records, including length of operation, amount of bleeding, complications, and cost of operation. The participants will be followed up for three times at 3, 6, and 12 months after the operation. The timeline of this study is shown in Fig. 1. The baseline information mainly includes the following:

1. Seizure condition: seizure frequency (time/month), seizure duration, severity, etc.
2. Neurological impairment: neurological function physical examination and National Institutes of Health Stroke Scale (NIHSS) score.
3. Use of anti-seizure medications: type and dosage of drugs.
4. Preoperative EEG examination: scalp EEG spectrum distribution, EEG Epileptogenic Index, etc.
5. Preoperative MRI.
6. Quality of life: using the Quality of Life in Epilepsy-89 scale for adults (ages 17–60) and the Quality of Life in Epilepsy-48 scale for children (ages 14–16). Patients not in the above age range will not be assessed for quality of life.
7. Cognitive function: using the Chinese version of the Wechsler Intelligence Scale-IV and the Chinese version of the Wechsler Development Scale-IV.

### Participant selection

The study is expected to enroll 240 participants. All participants will be recruited from the outpatient clinics of three centers and will be fully explained at the time of recruitment and will sign a written informed consent form. Minor participants and their legal guardians will also be fully explained about the study, and the written informed consent will be signed by their legal guardians.

Patients will be eligible for inclusion in the study only if all of the following criteria are applied:

1. Age ≥ 3 years at enrollment
2. Meets the ILAE guidelines for the diagnosis of drug-resistant epilepsy with persistent disabling seizures (at least 1 or more per month); seizures persist after 2 years of standardized treatment with at least one or more first-line antiepileptic drugs
3. With a definite epileptogenic zone by preoperative comprehensive evaluation, which belonged to focal epilepsy, and are suitable for stereotactic RF-TC, stereotactic LITT, or resective surgery
4. Ability to understand and speak Mandarin Chinese

Patients with the following conditions will be excluded:

Epileptogenic foci cannot be accurately located or multiple epileptogenic foci are suspected after a comprehensive evaluation, which are not suitable for the operation included in this study.

1. Pregnant or breastfeeding
2. Contraindications to surgery
3. History of previous operation for the purpose of treating epilepsy
4. Inclusion in any other clinical trial
5. Other conditions that the researcher thinks he/she is not suitable to participate in the study

### Procedures for withdrawal or discontinuation

Participants can withdraw at any time. Eligible participants will receive three follow-up visits at 3, 6, and 12 months after surgery or until any of the following events occur.

1. Death
2. End of the study period
3. Loss to follow-up: defined as referral to an epilepsy center that is not included in the study, or refusal to continue follow-up, or cannot be contacted by phone calls or WeChat for at least three times.
4. Other epilepsy surgical treatment is required due to the condition
5. Investigator considers it necessary for the participant to terminate the study

### Randomization and blinding

Randomization will be performed by independent investigators with the SAS (9.3, SAS Institute Inc., Cary, NC, USA) software. The resulting list of random numbers has been placed into sequentially numbered, sealed, opaque envelopes. Based on the information obtained from the envelopes, consecutively enrolled participants will be randomly assigned, in a 1:1 ratio, to stereotactic lesioning or classical epileptogenic zone resective surgery. Patients who are assigned to stereotactic lesioning surgery will be also assigned in a 4:1 ratio to a subgroup of RF-TC and a subgroup of LITT. The same group of neurosurgeons at different centers (three groups in total) will be responsible for different procedures for each participant.

Researchers and clinical research coordinators participate in the grouping process. Clinical research coordinators are responsible for informing patients, but they are not involved in the patient assessment and treatment process. All subjects and clinicians will be aware of the trial group assignments before surgical treatment, outcome assessors. However, outcome assessors and statisticians will not be aware of the subgroups of subjects

### Interventions

All three groups of participants will receive routine epilepsy care and antiepileptic drugs throughout the study. Participants will regularly take ASMs, which will be tailored according to the patient’s age, seizure type, drug interactions, physical health, and other factors. Medication adjustments will be handled by two experienced senior neurologists. Participants will be asked not to adjust the type and dose of medication and not to undergo other procedures throughout the observation period, unless there is a sudden exacerbation and they withdraw from the study. The surgical technique for LITT, RF-TC, and resective surgery is similar to that described previously [20, 24–27]. The main adverse events of the study will be mainly related to perioperative complications. Necessary medications and surgical procedures will be employed to prevent such damages. There is no anticipated additional harm and compensation for trial participation.

#### Arm 1: Resective surgery group

The resective surgery that patients will undergo in this study means the resection of epileptogenic foci. Patients with temporal lobe epilepsy will undergo standard anterior temporal lobectomy, and those with extratemporal lobe epilepsy will undergo lesionectomy and corticectomy with different lobes. The surgical techniques are roughly based on the previous research related to the resective surgery [28–30]. Surgical procedures for all patients will be recorded by the operating neurosurgeon.

#### Arm 2: Stereotactic EEG-guided RF-TC group

Patients enrolled in the stereotactic EEG-guided RF-TC group will undergo multicontact electrodes (RSDE-16/RSDE-12, Sinovation, China) implantation. After the intracranial electrodes are implanted, additional CT scans will be obtained to confirm their locations. Intracranial EEG recordings will be monitored to locate the epileptogenic zone. RF-TC will be performed on the contacts of the epileptogenic zone using a radiofrequency lesion generator (model R-2000B M1, BNS, China). A test heating will be performed first, and the patient will be monitored by EEG for 1 day. If the patient does not experience any unacceptable complications, RF-TC will be proceeded (3 W, 120 s). After RF-TC, the patient will be monitored by EEG for at least 1 night. The electrodes will then be removed, and the skin will be closed.

#### Arm 3: MRI-guided stereotactic LITT group

After local anesthesia, skull markers will be implanted and then a CT scan will be performed, and the results of which will be fused with T1-weighted MRI by the Sinoplan (V2, Sinovation, China) software for robot navigation registration. After general anesthesia, the patient’s head will be fixed with a magnetic resonance compatible head frame and connected to the operating table and SR1 stereotactic robot (Sinovation, China). After registering the surgical plan, the surgical field will be sterilized, the skull will be drilled, the dura will be punctured, and a probe will be used to ensure smooth access to the fiber-optic cannula. The guide screw will be installed, the cooling jacket containing the fiber will be placed at the planned depth, and an MRI scan will be performed to determine the location of the fiber. After confirming that the position of the optical fiber is in line with the plan, a series of ablations will be performed with real-time visualization using MRI thermometry to ensure that target structures reached temperatures between 60 and 80 °C. If necessary, the optical fiber will be withdrawn for repeated ablation. After all ablations are completed, the scope of ablation will be determined by scanning enhanced nuclear magnetic resonance. Then, the laser fiber, cooling catheter, and anchor bolt will be removed, the skin will be closed, and the patient will be awakened.

### Outcomes

#### Primary outcome

The primary outcome is the participant’s seizure remission rate, which was defined by the following formula:

$$\mathrm{Seizure\,remission\,rate}=\frac{\mathrm{Baseline\,seizure\,frequency}(\mathrm{per\,month}) -\mathrm{ Post}-\mathrm{op\,seizure\,frequency }(\mathrm{per\,month})}{\mathrm{Baseline\,seizure\,frequency}(\mathrm{per\,month})}\times 100\%$$

#### Secondary outcomes

1. Engel classification at one-year follow-up after surgery.
2. The proportion of participants with neurologic impairment after surgery, primarily determined by the change in NI.SHH scale score at each post-operative visit and neurological physical examination findings at each post-operative visit.
3. Spectrum distribution and Epileptogenic Index change of video EEG at each post-operative visit.
4. Participants’ quality of life after treatment. Evaluation will be performed at each post-operative visit by the Quality of Life in Epilepsy-89 scale (adults > 17 years) and the Quality of Life in Epilepsy-31 scale (children 4–16 years).
5. Epilepsy-related medical costs at each post-operative visit.

### Statistical analysis

Statistical analysis will be mainly performed by researchers from Sanbo Brain Hospital, Capital Medical University, using the SAS 9.3 (SAS Institute Inc., Cary, NC, USA) software. Take the test level *α* = 0.05. The Kolmogorov-Smirnov test will be used to test whether the variables will be normally distributed. Measurement data conforming to normal distribution will express as mean ± standard deviation (‾X ± s), and measurement data not conforming to normal distribution will be expressed as interquartile range *M* (Q25, Q75). The *t*-test will be used to compare the normal distribution of continuous variables. The rank sum test will be used to compare the continuous variables that do not conform to the normal distribution. The chi-square test will be used to compare the categorical data. Univariate and multivariate methods will be used to analyze the preoperative and operative factors associated with seizure-free. Analysis of the primary outcome will be by intention-to-treat.

### Sample size

According to earlier studies and the previous work of our research team, the effective rates of stereotactic EEG-guided RF-TC, MRI-guided stereotactic LITT, and resective surgery are similar, and the rates of postoperative neurological impairment are 2%, 2%, and 11%. We expect to use a one-sided 95% confidence interval, a noninferiority limit of 10%, and an expected withdrawal rate of 20%. Therefore, we expect to enroll a total of 240 patients in this study. Patients will be recruited from outpatient and MDT with strict inclusion criteria.

### Data management

All baseline information and outcome-related data will be evaluated and collected by two experienced clinicians (one neurologist and one neurosurgeon). Detailed data collection and follow-up time are shown in Table 1. Each patient’s data will be completed into an electronic case report form (eCRF) by an independent clinical research coordinator. Participants will be given trial information to improve participation. In order to ensure the completion of the follow-up, we will contact participants by phone in advance.

**Table 1:** Participant timeline

**Study period**	**Enrollment**	**Allocation**	**Operation**	**Follow-up**	**Follow-up**	**Follow-up**	**Follow-up**
**Time point**	− 10 days	− 7 days	0	7 ± 2 days	90 ± 7 days	180 ± 14 days	365 ± 14 days
**Participants**							
Eligibility screen	X						
Informed consent	X						
Randomization	X						
Demography		X					
**Interventions**							
MRI-guided stereotactic LITT group			X				
Stereotactic EEG-guided RF-TC group			X				
Resective surgery group			X				
**Outcomes**							
Seizure remission rate					X	X	X
Engel classification							X
ASMs		X		X	X	X	X
Neurological function examination		X		X	X	X	X
Scalp EEG		X			X	*X	X
NISHH		X					X
WAIS-IV-C/WCIS-IV-C		X					X
WADS-IV-C/WCDS-IV-C		X					X
QOILE-89/QOILE-48		X					X
PET		*X					*X
MRI		X			X	*X	X
Operative information			X				X
Adverse events				X	X	X	X

*X*, required; *X, optional; *LITT* Laser interstitial thermal therapy, *EEG* Electroencephalography, *RF-TC* Radiofrequency thermocoagulation, *ASM* Anti-seizure medications, *WAIS-IV-C* Chinese version of the Wechsler Adult Intelligence Scale-IV, *WCIS-IV-C* Chinese version of the Wechsler Children’s Intelligence Scale-IV, *WAIS-IV-C* Chinese version of the Wechsler Adult Development Scale-IV, *WCIS-IV-C* Chinese version of the Wechsler Children’s Development Scale-IV, *QOLIE-89* Quality of Life in Epilepsy-89, *QOLIE-48* Quality of Life in Epilepsy-48, *PET* positron emission tomography, *MRI* Magnetic resonance imaging

All related data will be collected and entered into an eCRF and study database. The investigators and clinical coordinators will be responsible for ensuring that the required data are collected and entered into the eCRF. The original information will be kept in paper medical records for data traceability purposes. The eCRF will be printed as a paper version and stored in a dedicated locked filing cabinet.

Only authorized researchers will be allowed to process the research data under computer password protection. The final data will be anonymized by completely removing any participant’s identifying information.

### Quality control

The independent trial monitoring committee is responsible for quality control and interim analyses. The trial monitoring committee usually checks the status of the trial at the three centers on a monthly basis and presents the quality control results at a joint meeting that includes the investigators and clinical research coordinators. Quality control activities include the following:

1. Request site support for study monitoring activities.
2. Conduct unified training and assessment for the core research personnel of each center before the study begins.
3. The study medical records and eCRF of the participants will be checked to confirm that the research team adhered to the protocol and that the data will be accurately recorded in the eCRF.
4. Check that the completed eCRF is complete, of good quality, and properly stored.

If the monitor suspects that the study may not conform to the best protocol, specific measures should be taken to assess the situation, identify problems, and implement specific action plans to correct the situation. The trial is independently audited annually by the Beijing Municipal Health Commission.

### Ethics and communication

This study protocol has been approved by the Ethics Committee of Sanbo Brain Hospital, Capital Medical University (project number: SBNK-YJ-2022-006-01).

Modifications to the study protocol will be reviewed by the ethics committee. Important protocol modifications will be communicated to the Beijing Municipal Health Commission for approval before implementation. Written informed consent will be obtained from all participants before any patient data or patient information is collected. The progress and results of this study will be made publicly available on the ClinicalTrials.gov website. The results of this study will be disseminated through peer-reviewed publications and conference presentations. Authorship eligibility guidelines will be followed.

## Discussion

The randomized controlled trial can minimize the possible biases in the design and conduct of clinical trials. The conclusion of a well-designed and rigorously conducted randomized controlled trial is recognized as high-level evidence. To date, we have found no reports in the literature of randomized clinical trials of LITT and RF-TC for the treatment of focal DRE.

Epileptogenic focus resection is currently the most common and effective surgical treatment for focal DRE and is more likely to mean a radical cure. Among them, temporal lobectomy is the most common and has the best prognosis, while frontal lobectomy is the second most common [31–33]. Two randomized clinical trials of temporal lobe epilepsy found that patients treated with surgery were more likely to be seizure-free than those who continued medical therapy [28, 34]. However, it has been reported that resective surgery may lead to hemiplegia, memory impairment, visual field defect, and other complications [35, 36].

By selectively damaging a limited volume to the potential seizure initiation zone, stereotactic lesioning can limit the risk of neurologic impairment after surgery. However, despite the promising results, the seizure outcome after RF-TC and LITT is still not fully understood, with meta-analyses showing that seizure-free and responder rates varied widely across studies [25, 37, 38]. What is more, none of the studies included in the meta-analysis compared stereotactic injury with resective surgery. In addition, the predictors associated with postoperative outcomes remain unclear. Although some studies suggest that specific patient populations may achieve better outcomes, such as RF-TC-treated patients with periventricular nodular ectopia and LITT-treated patients with hypothalamic hamartoma. But to date, there have been few studies on the impact of the underlying etiology on the success rate of stereotactic lesioning. As mentioned above, there are still some deficiencies in the current research on stereotactic lesioning, which are the key issues of this study.

The protocol for this study was developed after careful discussion by MDT physicians in neurology, neurosurgery, neuroimaging, and neuropsychology. There was no patient or public involvement in the design of our study. We will do our best to perform our duties to provide reliable evidence for stereotactic lesioning in the treatment of epilepsy.

## Trial status

Recruitment ongoing (the first participant recruited on 20 September 2022, approximate recruitment completed date: December 31, 2023).

The protocol version is 1.0, and the version date is June 1, 2022.

## Data Availability

The progress and results of this study will be updated on the ClinicalTrials.gov website. The data set for this study could be obtained from the corresponding author after the study is completed for reasonable reasons. The full protocol can be shared with no restrictions on request.

## Abbreviations

RF-TC: Radiofrequency thermocoagulation
LITT: Laser interstitial thermal therapy
SEEG: Stereotactic electroencephalography
ASMs: Anti-seizure medications
DRE: Drug-resistant epilepsy
MRI: Magnetic resonance imaging
NISHH: National Institutes of Health Stroke Scale
EEG: Electroencephalogram
eCRF: Electronic case report form
